# Variability in Total Cholesterol Concentration Is Associated With the Risk of Dementia: A Nationwide Population-Based Cohort Study

**DOI:** 10.3389/fneur.2019.00441

**Published:** 2019-05-07

**Authors:** Hye Soo Chung, Ji Sung Lee, Jung A. Kim, Eun Roh, You Bin Lee, So Hyeon Hong, Nam Hoon Kim, Hye Jin Yoo, Ji A. Seo, Sin Gon Kim, Nan Hee Kim, Sei Hyun Baik, Kyung Mook Choi

**Affiliations:** ^1^Division of Endocrinology and Metabolism, Department of Internal Medicine, College of Medicine, Hallym University, Seoul, South Korea; ^2^Clinical Research Center, Asan Medical Center, College of Medicine, Ulsan University, Seoul, South Korea; ^3^Division of Endocrinology and Metabolism, Department of Internal Medicine, College of Medicine, Korea University, Seoul, South Korea

**Keywords:** variability, total cholesterol, dementia, Alzheimer's disease, vascular dementia

## Abstract

**Introduction:** Although total cholesterol (TC) variability is suggested as a risk factor for cardiovascular and cerebrovascular disease, there is no previous study to evaluate the association between TC variability and the development of dementia.

**Methods:** Using the Korean National Health Insurance Service–Health Screening Cohort (NHIS-HEALS), the main outcomes were newly diagnosed all-cause dementia, Alzheimer's disease (AD), or vascular dementia (VaD) between January 1, 2008, and December 31, 2015. Visit-to-visit TC variability was measured as variability independent of the mean (TC-VIM), coefficient variance (TC-CV), and standard deviation (TC-SD).

**Results:** In a total of 131,965 Koreans, there were 3,722 all-cause dementia (2.82%), 2,776 AD (2.10%), and 488 VaD (0.37%) during the median follow-up of 8.4 years. Kaplan–Meier curves showed increased cumulative incidences for all in the group of the highest quartiles of TC variability compared to the others. Regression using the Fine and Gray hazards model showed a steadily increasing risk of all-cause dementia with higher quartiles of TC variability. After adjusting for confounders including mean TC level and comparing the highest and lowest TC-VIM quartiles, the hazard ratios (HRs) for all-cause dementia and AD were 1.15 [95% confidence interval (CI) = 1.05–1.27; *P* = 0.003] and 1.12 (95% CI = 1.00–1.25; *P* = 0.040), respectively. The incidence of VaD was not significantly higher in the higher-quartile groups compared to that in the lowest-quartile group in TC-VIM variability (HR 1.22; 95% CI = 0.95–1.59; *P* = 0.122). These associations were consistent with TC variability defined by TC-CV or TC-SD.

**Conclusions:** For the first time, we have demonstrated that a higher visit-to-visit variability in TC independent of mean TC is associated with an increased risk of all-cause dementia and AD in the general population.

## Introduction

With increasing numbers of elderly individuals in the population, the prevalence of dementia is also gradually increasing. The worldwide burden of dementia is presumed to be 5–7% among those over 60 years of and 80% among those over 90 years (1). In Korea, the disability-adjusted life years of dementia per 100,000 people in 2008 was 528 person-years overall and 5,117 person-years among those 65 years and older, and the burden of dementia is expected to increase steeply (2). Therefore, communities are encountering this growing burden, and caregivers must provide long-term care for patients with dementia (3).

Numerous studies have reported that visit-to-visit variability in cardiovascular risk factors is independently associated with the development of cognitive dysfunction or dementia. In a prospective study including 5,461 participants over 70 years of age, a higher visit-to-visit variability of blood pressure was associated with cortical infarcts, lower hippocampal volume, and impaired cognitive function even after adjusting for mean blood pressure (4). In a three-city study, higher visit-to-visit variability in systolic blood pressure was significantly related to an increased risk of Alzheimer's disease (AD) (5). Additionally, Lattanzi et al. showed that AD patients had an increased coefficient of variation (CV) and standard deviation (SD) in both systolic and diastolic blood pressure compared to those of healthy controls (6). Moreover, Nagai and Kario suggested that higher visit-to-visit variability in blood pressure was significantly associated with cognitive impairment, vascular dementia (VaD), and AD through the pathophysiology of arterial remodeling, amyloid β-peptide deposition, silent cerebral injury, and dysregulated cerebral circulation (7). Another study of 311 community-dwelling women older than 65 years of age reported that reduced heart rate variability reflecting cardiac autonomic dysfunction was correlated with impaired cognitive function (8). In a Taiwan diabetes study cohort including 16,706 patients with type 2 diabetes, increased glycemic variability measured as fasting plasma glucose (FPG)-CV and glycated hemoglobin-CV was related to high risks of AD (9). Finally, a cross-sectional study reported that higher low-density lipoprotein cholesterol (LDL-C) variability was related to lower cognitive function in older participants with a high risk of vascular disease (10).

Nevertheless, to the best of our knowledge, no study has assessed the association between long-term total cholesterol (TC) variability and dementia. Therefore, the present study examined the relationships between visit-to-visit TC variability and the incidence of dementia, including AD and VaD, using the longitudinal National Health Insurance Service–National Health Screening Cohort (NHIS-HEALS) database.

## Materials and Methods

### Data Sources

**Figure 1:**
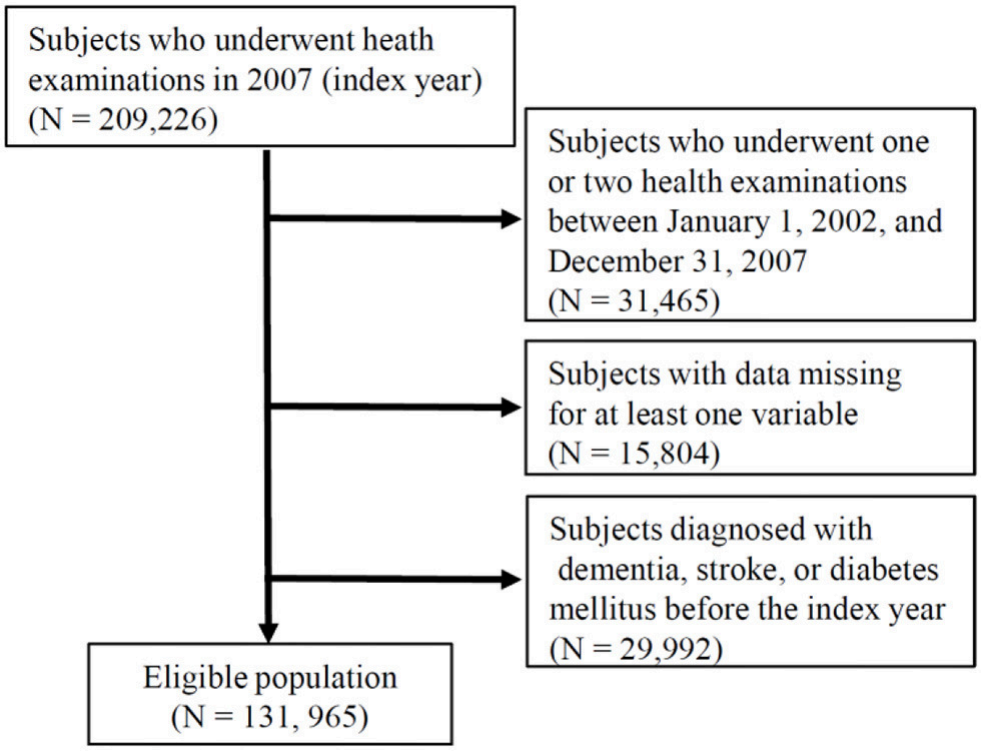
Flowchart of the study population. NHIS-HEALS, National Health Insurance Service–National Health Screening Cohort.

The NHIS in the Republic of Korea is a government-operated compulsory social health insurance program that includes nearly all citizens (about 98%), who are recommended to receive standardized national health examination programs biannually (annually for manual workers) (11, 12). All health examinations such as anthropometric measurement and laboratory tests are conducted after an overnight fast, and the Korean Association of Laboratory Quality Control superintends the quality control procedures. The NHIS-HEALS comprises a randomly sampled database of ~10% of all participants, aged between 40 and 79 years, within the NHIS data. This study consisted of individuals who underwent the national health examinations in 2007 (the index year) and three or more health examinations between January 1, 2002, and December 31, 2007. After excluding individuals with missing data in at least one variable and who had a previous diagnosis of dementia, stroke, or diabetes mellitus before 2007, a total of 131,965 individuals were finally included in the analysis (Figure 1). Informed consent was waived because anonymous and de-identified information was used for analysis. These protocols were approved by both the NHIS review committee and the institutional review board (IRB). The Korea University IRB approved the study protocol in accordance with the Declaration of Helsinki of the World Medical Association.

### Measurements and Definitions

Body mass index (BMI) was computed as weight (kilograms)/height squared (meters^2^). The smoking and alcohol drinking statuses were obtained from responses to the health examination questionnaire. Regular exercise was defined as strenuous physical activity for at least 20 min and ≥n times/week. Income level was dichotomized at the lower 10%. The presence of diabetes was defined based on the criterion of FPG level ≥7 mmol/L or the presence of at least one claim per year for the prescription of antidiabetic medication under the International Classification of Diseases, Tenth Revision (ICD-10), codes (E10–E14). The presence of hypertension was defined based on the criterion of systolic/diastolic blood pressure ≥140/90 mmHg or the presence of at least one claim per year for the prescription of an antihypertensive agent under ICD-10 codes (I10–I15). The presence of dyslipidemia was defined based on the criterion of TC level ≥6.2 mmol/L or the presence of at least one claim per year for the prescription of a lipid-lowering agent under ICD-10 code (E78). Diagnosis of stroke was defined as ICD-10 codes (I60–I64) on the admission record with computerized tomography or magnetic resonance imaging claim data. Diagnosis of myocardial infarction (MI) was defined as ICD-10 codes (I21–I22) during hospitalization or these codes having been recorded at least two times.

### Definition of Total Cholesterol Variability

The TC variability was determined from at least three measurements of TC values during health examinations: three measurements (*n* = 67,609, 51%), four measurements (*n* = 14,069, 11%), five measurements (*n* = 18,840, 14%), and six measurements (n = 31,447, 24%). For descriptive TC variability, we used three indices of variability: TC—variability independent of the mean (VIM), TC—CV, and TC—SD. VIM was defined as 100 × SD/Meanβ, where β is the regression coefficient, based on the natural logarithm of the SD over the natural logarithm of the mean. CV was defined as SD/mean × 100 (%).

### Study Outcomes

We examined newly diagnosed all-cause dementia, AD, and VaD as primary outcomes between January 1, 2008, and December 31, 2015. Diagnosis of all-cause dementia was defined based on the first prescription of an anti-dementia drug [acetylcholinesterase inhibitors (donepezil, galantamine, rivastigmine) or N-methyl-D-aspartate (NMDA) receptor antagonist (memantine)] under ICD-10 codes (F00–F03, G30) among outpatients or hospitalized patients. Diagnosis of AD, the most common subtype of dementia, was defined based on the first prescription of an anti-dementia drug with an ICD-10 code for AD (F00, G30), and the diagnosis of VaD was defined based on the first prescription of an anti-dementia drug with an ICD-10 code for VaD (F01). In Korea, in order to prescribe anti-dementia drugs, the Korea National Health Insurance Reimbursement Criteria require that physicians record evidence of cognitive dysfunction: (a) Mini-Mental State Examination (MMSE) ≤ 26 and either (b) Global Deterioration Scale (GDS) ≥3, or (c) Clinical Dementia Rating (CDR) ≥1.

### Statistical Analysis

Baseline characteristics are presented as means ± SDs for continuous variables and as percentages for categorical variables. Participants were classified into quartiles according to the TC variability. Differences between groups were identified by analysis of variance (ANOVA) for continuous variables and χ^2^-tests for categorical variables. Kaplan–Meier curves of cumulative incidence for all-cause dementia were produced for the four quartile groups of TC variability. The hazard ratios (HRs) and 95% confidence interval (CI) values for all-cause dementia, AD, and VaD were analyzed using the method of Fine and Gray, in order to take into account the competing risk of mortality (13), for the quartile groups of TC variability, adjusted for age, sex, BMI, alcohol consumption, smoking, regular exercise, income, hypertension, dyslipidemia, history of MI, and mean TC. Cumulative incidence functions (CIFs) were used to estimate the probability of the occurrence of all-cause dementia, AD, and VaD. We applied the proportional hazards model for the subdistribution of a competing risk to estimate the subdistribution HR and 95% CI.

We also performed subgroup analyses of the association between TC-VIM variability and incidence of all-cause dementia, AD, or VaD by age, sex, BMI, hypertension, use of an antihypertension medication, dyslipidemia, use of a lipid-lowering agent, history of MI, current smoking, and income. In subgroup analyses, the HR and 95% CI of the group of the upper three quartiles (Q2–Q4) were compared with those of the lowest quartile (Q1) as the reference group using cox proportional hazards regression analyses with interaction effect. Because of *post hoc* subgroup analyses, we did not adjust for multiple testing.

All statistical results were analyzed using SAS 9.4 (SAS Institute Inc., Cary, NC, USA), and *P*-values < 0.05 were assumed to indicate statistical significance.

## Results

### Baseline Characteristics of the Study Population

Table 1 describes the characteristics of the study participants according to the VIM quartiles for TC variability. The ranges of the VIM quartiles with TC variability were 8.40 ± 2.77%, 15.00 ± 1.59%, 21.05 ± 2.06%, and 34.82 ± 10.51%, respectively (*P*-value < 0.001). The higher-quartile groups of TC variability were older and had higher proportions of women and lower income compared to the lower-quartile group. The prevalence of comorbid conditions such as hypertension, dyslipidemia, and MI also increased incrementally according to quartiles of TC variability. Expectably, BMI, systolic blood pressure, diastolic blood pressure, FPG level, TC level, and proportion of antihypertensive or lipid-lowering agent use were higher in the higher-quartile groups of TC variability. Similar relationships in the baseline characteristics were observed in the quartiles of TC-CV (Supplementary Table 1) and TC-SD (Supplementary Table 2).

**Table 1:** Baseline characteristics of the subjects according to the total cholesterol (TC) variability measured as variability independent of the mean (VIM).

	**Q1**	**Q2**	**Q3**	**Q4**	***P-*value**
*N*	32,991	32,991	32,992	32,991	
Age (years)	55.5 ± 8.7	54.5 ± 8.2	54.9 ± 8.4	56.9 ± 8.9	< 0.001
Sex (male) (*n*, %)	18,724 (56.8)	20,140 (61.0)	19,063 (57.8)	16,900 (51.2)	< 0.001
Body mass index (kg/m^2^)	23.8 ± 2.8	23.8 ± 2.8	23.8 ± 2.8	24.0 ± 2.9	< 0.001
Systolic BP (mmHg)	124.3 ± 15.4	124.5 ± 15.3	124.9 ± 15.4	125.8 ± 15.9	< 0.001
Diastolic BP (mmHg)	77.6 ± 10.1	77.9 ± 10.1	78.1 ± 10.2	78.2 ± 10.2	< 0.001
AST (IU/L)	25.1 ± 11.6	25.4 ± 13.0	25.8 ± 15.3	27.1 ± 18.8	< 0.001
ALT (IU/L)	23.5 ± 15.5	24.2 ± 18.9	24.5 ± 17.9	25.7 ± 21.6	< 0.001
GGT (IU/L)	32.7 ± 36.6	35.1 ± 40.3	36.1 ± 42.9	39.2 ± 54.8	< 0.001
Fasting plasma glucose (mmol/L)	5.10 ± 0.63	5.10 ± 0.64	5.10 ± 0.65	5.13 ± 0.66	< 0.001
Mean TC (mg/dl)	194.9 ± 29.4	196.1 ± 29.2	198.2 ± 29.6	203.8 ± 31.4	< 0.001
TC variability					
VIM (%)	8.40 ± 2.77	15.00 ± 1.59	21.05 ± 2.06	34.82 ± 10.51	< 0.001
CV (%)	4.30 ± 1.45	7.65 ± 0.95	10.69 ± 1.24	17.44 ± 5.13	< 0.001
SD (IU/L)	8.29 ± 2.83	14.87 ± 2.03	20.99 ± 2.75	35.39 ± 12.07	< 0.001
Current smoker (*n*, %)	6,059 (18.4)	6,894 (20.9)	6,544 (19.8)	5,829 (17.7)	< 0.001
Alcohol consumption (*n*, %)	14,024 (42.5)	14,950 (45.3)	14,291 (43.3)	12,640 (38.3)	< 0.001
Regular exercise (*n*, %)	3,446 (10.4)	3,077 (9.3)	3,088 (9.4)	3,420 (10.4)	< 0.001
Income (lower 10%) (*n*, %)	2,299 (7.0)	2,309 (7.0)	2,555 (7.7)	2,871 (8.7)	< 0.001
Hypertension (*n*, %)	17,457 (52.9)	18,077 (54.8)	18,726 (56.8)	20,336 (61.6)	< 0.001
Dyslipidemia (*n*, %)	5,528 (16.8)	7,696 (23.3)	10,644 (32.3)	18,531 (56.2)	< 0.001
History of myocardial infarction (*n*, %)	182 (0.6)	154 (0.5)	202 (0.6)	430 (1.3)	< 0.001
Use of antihypertensive agent (*n*, %)	10,170 (30.8)	9,776 (29.6)	10,536 (31.9)	13,596 (41.2)	< 0.001
Use of lipid-lowering agent (*n*, %)	2,487 (7.5)	2,677 (8.1)	3,530 (10.7)	8,054 (24.4)	< 0.001

*AST, aspartate transaminase; ALT, alanine transaminase; BP, blood pressure; GGT, γ-glutamyl transferase; VIM, variability independent of the mean; CV, coefficient of variation; SD, standard deviation*.

*P-value by ANOVA and chi-square test. Data are expressed as mean ± SD, or n (%)*.

### Implication of Total Cholesterol Variability With All-Cause Dementia, Alzheimer's Disease, and Vascular Dementia

**Figure 2:**
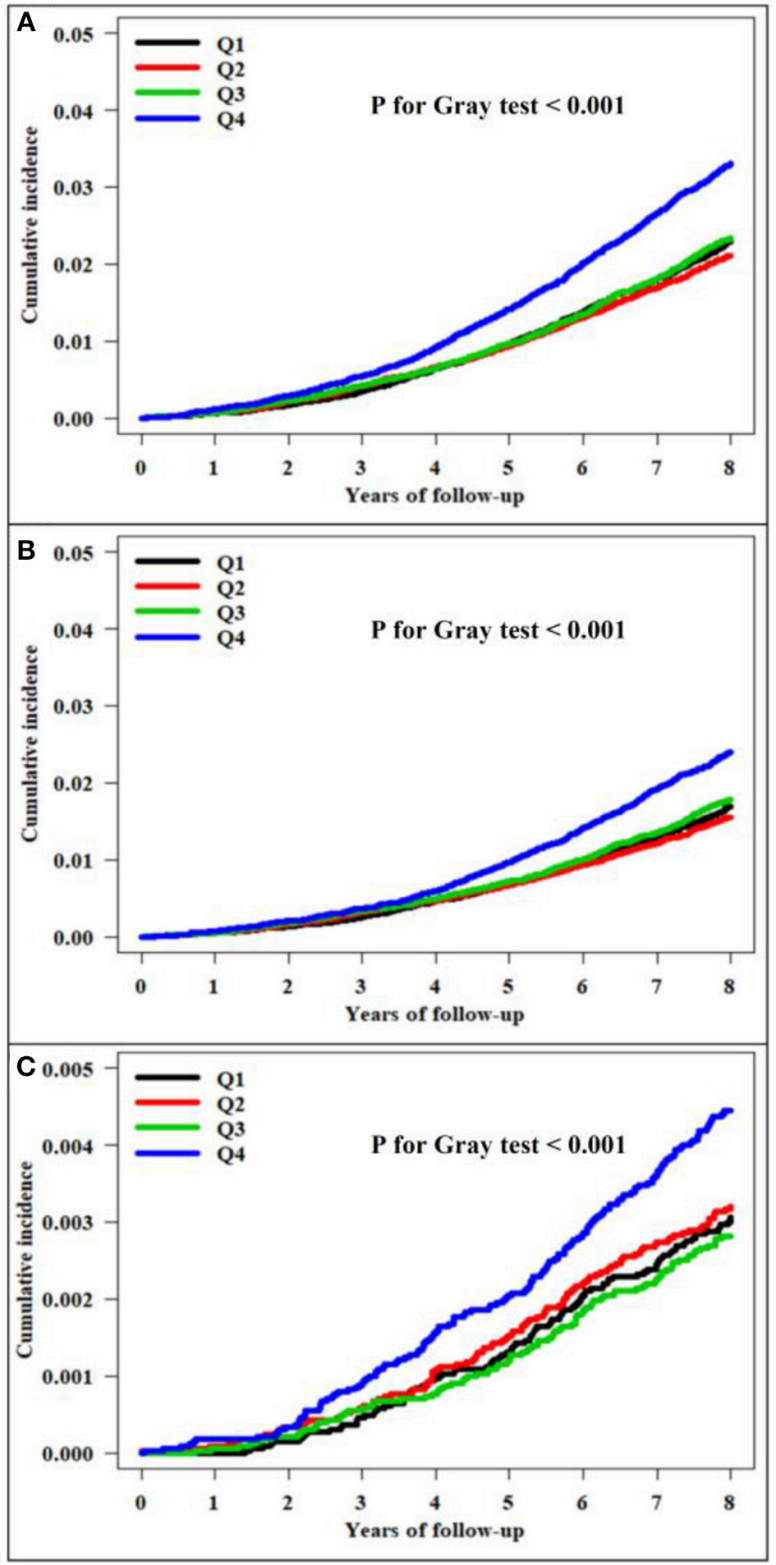
Kaplan–Meier estimates of the cumulative incidence by quartile of total cholesterol variability measured as the variability independent of the mean (VIM). **(A)** All-cause dementia. **(B)** Alzheimer's disease. **(C)** Vascular dementia.

There were 3,722 all-cause dementia (2.82%), 2,776 AD (2.10%), and 488 VaD (0.37%) during the median follow-up of 8.4 years in the entire cohort. The unadjusted cumulative incidences for all-cause dementia, AD, and VaD showed significantly higher incidences among the subjects in the fourth quartile of TC variability compared with those in the first quartile, measured as VIM (Figure 2), CV (all-cause dementia: *P* for Gray test < 0.001, AD: *P* for Gray test < 0.001, VaD: *P* for Gray test < 0.001), and SD (all-cause dementia: *P* for Gray test < 0.001, AD: *P* for Gray test < 0.001, VaD: *P* for Gray test = 0.005). The Fine and Gray hazards regression models revealed a steadily higher risk of all-cause dementia in the higher quartiles compared with that of the lowest-quartile group in TC variability (Table 2). In TC variability as measured by VIM, CV, and SD, the HRs for incident all-cause dementia were 1.15 (95% CI = 1.05–1.27; *P* = 0.003), 1.20 (95% CI = 1.09–1.32; *P* < 0.001), and 1.12 (95% CI = 1.02–1.23; *P* = 0.023) in the group of the highest quartile of TC variability compared to those of the lowest-quartile group after adjusting for multiple variables and the mean TC. Furthermore, TC variability was also identified as a meaningful predictor of AD, even after adjusting for confounding factors (Table 3). In TC variability as measured by VIM, CV, and SD, the adjusted HRs for incident AD were 1.12 (95% CI = 1.00–1.25; *P* = 0.040), 1.16 (95% CI = 1.04–1.29; *P* = 0.009), and 1.10 (95% CI = 0.98–1.23; *P* = 0.094) in group of the highest quartiles of TC variability compared to that of the lowest-quartile group. However, the risk of VaD was not significantly increased in the higher-quartile groups compared to that of the lowest-quartile group in TC variability as measured by VIM, CV, and SD [1.22 (95% CI = 0.95–1.57; *P* = 0.122), 1.34 (95% CI = 1.04–1.73; *P* = 0.023), and 1.12 (95% CI = 0.87–1.45; *P* = 0.374)] (Table 3).

**Table 2:** Hazard ratios (HRs) and 95% confidence intervals (CIs) of all-cause dementia by quartiles of total cholesterol (TC) variability.

	**Events (*n*)**	**Follow-up duration (person-years)**	**Incidence rate (per 1,000 person-years)**	**Adjusted HR (95% CI)**	***P*-value**
**TC Variability (VIM)**					
Q1	853	271,508	3.14	1 (ref)	
Q2	787	271,396	2.90	1.12 (1.02–1.24)	0.020
Q3	865	271,030	3.19	1.13 (1.03–1.25)	0.012
Q4	1,217	268,717	4.53	1.15 (1.05–1.27)	0.003
P for trend	0.004				
**TC variability (CV)**					
Q1	848	271,568	3.12	1 (ref)	
Q2	822	271,365	3.03	1.20 (1.09–1.32)	< 0.001
Q3	819	271,217	3.02	1.09 (0.99–1.20)	0.091
Q4	1,233	268,503	4.59	1.20 (1.09–1.32)	< 0.001
P for trend	0.002				
**TC variability (SD)**					
Q1	861	271,356	3.17	1 (ref)	
Q2	772	271,475	2.84	1.07 (0.97–1.18)	0.167
Q3	886	270,821	3.27	1.11 (1.01–1.22)	0.032
Q4	1,203	269,001	4.47	1.12 (1.02–1.23)	0.023
P for trend	0.018				

*VIM, variability independent of the mean; CV, coefficient of variation; SD, standard deviation*.

*Adjusted for age, sex, income, body mass index, hypertension, dyslipidemia, history of myocardial infarction, smoking, alcohol intake, exercise, and mean TC level*.

*Using regression methods of Fine and Gray for competing risk data (death is a competing event for dementia)*.

**Table 3:** Hazard ratios (HRs) and 95% confidence intervals (CIs) of Alzheimer's dementia and vascular dementia by quartiles of total cholesterol (TC) variability.

	**Events (n)**	**Follow-up duration (person-years)**	**Incidence rate (per 1,000 person-years)**	**Adjusted HR (95% CI)**	***P*-value**
**ALZHEIMER'S DISEASE**					
**TC variability (VIM)**					
Q1	632	271,508	2.33	1 (ref)	
Q2	591	271,396	2.18	1.14 (1.02–1.28)	0.020
Q3	664	271,030	2.45	1.17 (1.05–1.31)	0.005
Q4	889	268,717	3.31	1.12 (1.00–1.25)	0.040
P for trend	0.046				
**TC variability (CV)**					
Q1	633	271,568	2.33	1 (ref)	
Q2	617	271,365	2.27	1.21 (1.08–1.35)	0.001
Q3	630	271,217	2.32	1.12 (1.01–1.26)	0.040
Q4	896	268,503	3.34	1.16 (1.04–1.29)	0.009
P for trend	0.035				
**TC variability (SD)**					
Q1	633	271,356	2.33	1 (ref)	
Q2	585	271,475	2.15	1.11 (0.99–1.24)	0.084
Q3	664	270,821	2.45	1.12 (1.00–1.25)	0.041
Q4	894	269,001	3.32	1.10 (0.98–1.23)	0.094
P for trend	0.101				
**VASCULAR DEMENTIA**					
**TC variability (VIM)**					
Q1	109	271,508	0.40	1 (ref)	
Q2	114	271,396	0.42	1.21 (0.93–1.58)	0.147
Q3	101	271,030	0.37	1.00 (0.76–1.31)	0.988
Q4	164	268,717	0.61	1.22 (0.95–1.57)	0.122
P for trend	0.235				
**TC variability (CV)**					
Q1	104	271,568	0.38	1 (ref)	
Q2	119	271,365	0.44	1.33 (1.02–1.73)	0.034
Q3	91	271,217	0.34	0.94 (0.70–1.24)	0.645
Q4	174	268,503	0.65	1.34 (1.04–1.73)	0.023
P for trend	0.136				
**TC variability (SD)**					
Q1	116	271,356	0.43	1 (ref)	
Q2	102	271,475	0.38	1.02 (0.78–1.33)	0.884
Q3	115	270,821	0.42	1.06 (0.82–1.38)	0.658
Q4	155	269,001	0.58	1.12 (0.87–1.45)	0.374
P for trend	0.360				

*VIM, variability independent of the mean; CV, coefficient of variation; SD, standard deviation*.

*Adjusted for age, sex, income, body mass index, hypertension, dyslipidemia, history of myocardial infarction, smoking, alcohol intake, exercise, and mean total cholesterol level*.

*Using regression methods of Fine and Gray for competing risk data (death is a competing event for dementia)*.

**Figure 3:**
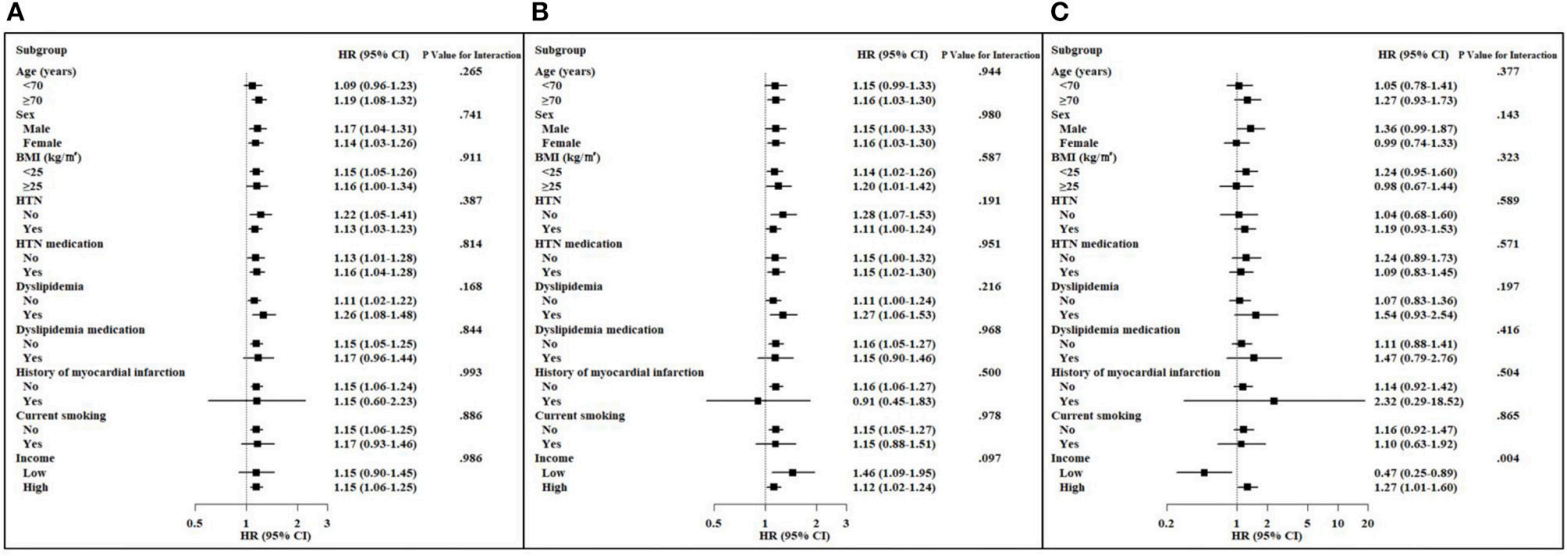
Hazard ratios (HRs) and 95% confidence intervals (CIs) of dementia in the highest three quartiles vs. the lowest quartile of total cholesterol variability (VIM) in subgroups. Adjusted for age, sex, body mass index (BMI), hypertension (HTN), use of antihypertension medication, dyslipidemia, use of a lipid-lowering agent, history of myocardial infarction, current smoking, and income. **(A)** All-cause dementia. **(B)** Alzheimer's disease. **(C)** Vascular dementia.

Subgroup analyses stratified by age, sex, BMI, hypertension, use of antihypertensive agents, dyslipidemia, use of lipid-lowering agents, history of MI, current smoking, and level of income were performed (Figure 3). The Q2–Q4 group of TC-VIM variability remained predictive of all-cause dementia in the subgroups of older (≥70 years) individuals, those without obesity, those not taking lipid-lowering agents, those without history of MI, non-smokers, and those with high income, irrespective of gender, history of hypertension and antihypertensive agents, and history of dyslipidemia, compared with the Q1 groups (Figure 3A). Additionally, the associations between TC-WIM variability and AD were consistent in subjects who were older (≥70 years), female, with or without obesity, without hypertension, using antihypertensive agents, with a history of dyslipidemia, not taking lipid-lowering agents, with no history of MI, and non-smokers, irrespective of income (Figure 3B). There was no statistically different incidence of all-cause dementia and AD within categories of several risk factors. A substantially higher risk of VaD was observed in the high-income subgroup compared to that of the low-income group (*P*-value for interaction = 0.004; Figure 3C).

## Discussion

Using a large-scale cohort data set with long follow-up duration, we demonstrated that high visit-to-visit TC variability was associated with the occurrence of all-cause dementia and AD independently of mean TC levels in the Korean population. However, there was no association between visit-to-visit TC variability and VaD. These results infer that variability in cholesterol level may be a novel predictor of upcoming dementia, including AD.

Variability in anthropometric or laboratory parameters may be surrogate markers for diverse diseases rather than measurement error. High intraindividual variability in cholesterol levels is linked to adverse outcomes. In the long-term Framingham Study including 2,912 men and women, high intraindividual variability in TC was related to increased risks of CVD and mortality (14). In addition, Kim et al. showed that high visit-to-visit variability in TC level is associated with the risk of stroke, MI, and mortality in Koreans (15). In the Treating-to-New-Targets (TNT) trial, Bangalore et al. reported that high LDL-C variability was also associated with stroke, MI, any coronary event, any cardiovascular event, and mortality after adjusting for mean LDL-C levels and statin treatment (16). In subjects with previous ST-segment elevation MI events, visit-to-visit LDL-C and high-density lipoprotein cholesterol (HDL-C) variability is an independent predictor of major adverse cardiovascular events including stroke and MI (17). Moreover, recent studies demonstrated that visit-to-visit variability in lipids is related to other chronic inflammatory outcomes such as the incidence of diabetes (18), diabetic nephropathy progression (19), and end-stage renal disease progression (20). Notably, Smit et al. reported that higher LDL-C variability, independent of statin use and mean LDL-C levels, is related to lower function in four cognitive domains, greater white matter hyperintensity volume, and lower cerebral blood flow (10). Similarly, positive correlations between LDL-C variability and dementia risk factors such as maximum carotid intima media thickness (21) or severity of obstructive sleep apnea (22) have been reported. Recently, using the data of NHIS, Lee et al. showed an association between increased variabilities in metabolic parameters, including blood pressure, glucose, cholesterol, and BMI, and risk of dementia during a median follow-up of 5.5 years (23). They concluded that there was a linear relationship between the number of increased variability parameters and risk of dementia. On the other hand, our study focused on the impact of higher visit-to-visit TC variability on long-term risk of dementia using the NHIS-HEALS database for a longer follow-up period (8.4 years). Therefore, we presented detailed results regarding the relationship between visit-to-visit TC variability and dementia, such as Kaplan–Meier curves by quartiles of TC variability (Figure 2) and HRs (95% CIs) in various subgroups of risk factors (Figure 3).

Several epidemiologic studies have revealed the relationship between TC level and the risk of cognitive outcomes. Although the relationship between hypercholesterolemia and impaired cognitive function was shown in animal studies (24–26), the findings were controversial and varied according to the study design in human studies. In a longitudinal population-based study, high TC level (≥6.5 mmol/L) or systolic blood pressure (≥160 mmHg) in midlife was predictive of an elevated the risk of AD during a mean 21 years of follow-up (27). However, another longitudinal study conducted over 32 years revealed that TC level in midlife was not significantly related to the risk of dementia (28). Additionally, Beydoun et al. used Cox proportional hazard models to show that increased first-visit TC level is associated with a decreased risk of mild cognitive impairment but not with dementia (29). A meta-analysis including these previous studies reported that high TC measured in midlife (40 < age ≤ 60 years) was significantly associated with an increased risk of late-life all-cause dementia (relative risk: 1.82; 95% CI: 1.27–2.60; *P* < 0.01) and AD (relative risk: 2.14; 95% CI: 1.33–3.44; *P* < 0.01) compared to individuals without high TC levels (30). Meanwhile, most studies that measured TC in late life have reported that high TC level is not associated with all-cause dementia (30–32) or AD (30–33). However, there is insufficient epidemiologic evidence to determine if high TC level is a predictable risk factor for dementia, including AD.

Although epidemiologic studies have assessed whether average TC level is a risk factor for cognitive outcomes, few have reported on serial changes in TC level. In a prospective study of 1,462 Swedish women, declining TC levels from midlife to late life were potentially related to an increased risk of AD (28). Beydoun et al. showed that a decline in TC level compared to baseline level was associated with an elevated risk of dementia among men but not women in subgroup analysis (29). Another study with 1,027 Japanese-American men reported that participants with dementia had sharply decreased serum TC levels in the early stages of dementia compared to those without dementia (34). Similarly, in Finnish men, the serial mean TC level in those with AD declined more drastically than that in the group without dementia (35). However, most previous studies on changes in TC focused on the decreasing trend of mean TC levels and dementia. To the best of our knowledge, the association between visit-to-visit variability in TC level and incident dementia or its subtypes has not been evaluated. The present study used nationwide population to confirm that visit-to-visit variability in TC level was independently associated with the risk of all-cause dementia and AD after adjusting for confounding factors.

The results of the present study showed that increased TC variability was not significantly associated with the risk of VaD. A possible reason for no association was the relatively younger age of the study population. Previous studies reported that the incidence of VaD increases exponentially as age increases after the age of 65 years (36, 37). Additionally, several previous studies demonstrated no meaningful associations between TC and VaD, concordant with our study findings. In a study of 1,027 Japanese-American men, Stewart et al. showed that a 26-year change in TC in midlife was not related to incident VaD (34). Another study in the general Japanese population older than 65 years of age reported that the risk factors for incident VaD included age, being an alcohol consumer, diabetes mellitus, stroke, blood pressure, and hematocrit, but not TC (38). In American community-based cohort studies of older age (>65 years), Reitz et al. reported that the risk of VaD was not related to TC, even though a weak relationship between VaD and subtypes of cholesterol including LDL cholesterol and HDL cholesterol was observed (33). Further investigation is necessary to confirm the association between TC variability and VaD, considering the relatively low incidence of VaD, as well as to understand the mechanisms.

Although the pathophysiological mechanisms of TC variability in dementia are not fully known, several pathways have been suggested. The predicted pathogenic role of variability in cholesterol levels is connected to inflammation and endothelial dysfunction, which results in the development of atherosclerosis (39). A previous study showed that increased levels of serum markers of endothelial dysfunction were associated with an increased risk of developing cognitive impairment (40). Another pathway contributing to dementia or cerebrovascular damage is plaque instability (41, 42), use of lipid-lowering medication, and non-adherence to the use of lipid-lowering medication (43). Animal studies have demonstrated that intermittent high-fat diets lead to atherosclerosis (44). Furthermore, in animal and human studies, lipid-lowering agents caused changes in the composition of atherosclerotic plaques and plaque rupture (45, 46). However, our study reported a consistent association between TC variability and all-cause dementia or AD in the subgroup without lipid-lowering medication. Additionally, there is a need to evaluate the cholesterol metabolism and metabolic products in the brain affected by TC level variability in the blood.

This study has several limitations. First, due to inherent limitations of the observational study design, we could not infer a causal relationship between visit-to-visit TC variability and dementia. Second, although we tried to eliminate the covariates known to affect the risk of dementia in our multivariate analyses, unmeasured confounding factors such as depression, cognition level, and education are possible. Third, the NHIS health examinations did not assess other lipid profile data including LDL-C, HDL-C, and triglyceride levels. Nevertheless, the present study has notable strengths, including the validation of a standardized database from the Korean government with detailed and credible information regarding medication usage and medical diagnosis. Moreover, this study had a sufficiently long median follow-up period of 8.4 years to evaluate the risk of dementia in the general population. Additionally, the variability in cholesterol levels was calculated using diverse indicators such as TC-VIM, TC-CV, and TC-SD, and their associations with dementia and its subtypes were similar and consistent.

In conclusion, we report for the first time that higher visit-to-visit TC variability is independently associated with an increased long-term risk of dementia and AD after adjusting for potential risk factors including mean TC. The present study using a nationwide population provides new insight into the relevance of the serial measurement of cholesterol levels in populations at high risk of dementia. Further replication studies in other age or ethnic groups are necessary to validate these findings.

## Contribution to the Field Statement

Previous studies have reported the relationship between TC level and the development of dementia. Nevertheless, no study has assessed the association between visit-to-visit variability in TC and dementia. In a longitudinal nationwide population-based cohort, we evaluated the relationships between visit-to-visit TC variability and the incidence of dementia, including AD and VaD. This study suggested that visit-to-visit TC variability, using three different indicators, is a risk factor for the development of all-cause dementia, and AD independently of other risk variables, including mean cholesterol. However, we showed that increased TC variability was not significantly associated with the risk of vascular disease. Further study is needed to replicate these findings in other age or ethnic groups and to elucidate the mechanism of TC variability in the pathogenesis of dementia or AD.

## Data Availability

The data that support the findings of this study are available from the corresponding author upon reasonable request and approval of the institutional review board.

## Ethics Statement

The Korea University IRB approved the study protocol in accordance with the Declaration of Helsinki of the World Medical Association.

## Author Contributions

HC, JL, and KC contributed to the study concept and design, contributed to the data acquisition, and conducted the manuscript drafting. HC, JL, JK, ER, YL, SH, HY, and KC contributed to the data analysis and interpretation. NHoK, JS, SK, NHeK, and SB performed critical manuscript revision.

### Conflict of Interest Statement

The authors declare that the research was conducted in the absence of any commercial or financial relationships that could be construed as a potential conflict of interest.

## References

[1] Prince M Bryce R Albanese E Wimo A Ribeiro W Ferri CP The global prevalence of dementia: a systematic review and meta-analysis. Alzheimers Dement. (2013) 9:63–75 e2. 10.1016/j.jalz.2012.11.007 23305823

[2] Park JH Eum JH Bold B Cheong HK. Burden of disease due to dementia in the elderly population of Korea: present and future. BMC Public Health. (2013) 13:293. 10.1186/1471-2458-13-293 23552095 PMC3626581

[3] Etters L Goodall D Harrison BE. Caregiver burden among dementia patient caregivers: a review of the literature. J Am Acad Nurse Pract. (2008) 20:423–8. 10.1111/j.1745-7599.2008.00342.x 18786017

[4] Sabayan B Wijsman LW Foster-Dingley JC Stott DJ Ford I Buckley BM. Association of visit-to-visit variability in blood pressure with cognitive function in old age: prospective cohort study. BMJ. (2013) 347:f4600. 10.1136/bmj.f4600 23900315

[5] Alperovitch A Blachier M Soumare A Ritchie K Dartigues JF Richard-Harston S. Blood pressure variability and risk of dementia in an elderly cohort, the Three-City Study. Alzheimers Dement. (2014) 10:S330–7. 10.1016/j.jalz.2013.05.1777 23954028

[6] Lattanzi S Viticchi G Falsetti L Buratti L Luzzi S Provinciali L. Visit-to-visit blood pressure variability in Alzheimer disease. Alzheimer Dis Assoc Disord. (2014) 28:347–51. 10.1097/WAD.0000000000000040 24731982

[7] Nagai M Kario K. Visit-to-visit blood pressure variability: a possible marker of cognitive decline in Alzheimer's disease?. Neurobiol Aging. (2015) 36:e1. 10.1016/j.neurobiolaging.2014.06.027 25085785

[8] Kim DH Lipsitz LA Ferrucci L Varadhan R Guralnik JM Carlson MC. Association between reduced heart rate variability and cognitive impairment in older disabled women in the community: women's health and aging study I. J Am Geriatr Soc. (2006) 54:1751–7. 10.1111/j.1532-5415.2006.00940.x 17087704 PMC2276586

[9] Li TC Yang CP Tseng ST Li CI Liu CS Lin WY. Visit-to-visit variations in fasting plasma glucose and HbA1c associated with an increased risk of Alzheimer disease: Taiwan Diabetes Study. Diabetes Care. (2017) 40:1210–7. 10.2337/dc16-2238 28705834

[10] Smit RA Trompet S Sabayan B le Cessie S van der Grond J van Buchem MA. Higher visit-to-visit low-density lipoprotein cholesterol variability is associated with lower cognitive performance, lower cerebral blood flow, and greater white matter hyperintensity load in older subjects. Circulation. (2016) 134:212–21. 10.1161/CIRCULATIONAHA.115.020627 27436880

[11] Kwon S. Thirty years of national health insurance in South Korea: lessons for achieving universal health care coverage. Health Policy Plan. (2009) 24:63–71. 10.1093/heapol/czn037 19004861

[12] Seong SC Kim YY Park SK Khang YH Kim HC Park JH. Cohort profile: the National Health Insurance Service–National Health Screening Cohort (NHIS-HEALS) in Korea. BMJ Open. (2017) 7:e016640. 10.1136/bmjopen-2017-016640 28947447 PMC5623538

[13] Fine JP Gray RJ A proportional hazards model for the subdistribution of a competing risk. J Am Stat Assoc. (1999) 94:496–509.

[14] Kreger BE Odell PM D'Agostino RB Wilson PF. Long-term intraindividual cholesterol variability: natural course and adverse impact on morbidity and mortality—the Framingham Study. Am Heart J. (1994) 127:1607–14. 8197990 10.1016/0002-8703(94)90393-x

[15] Kim MK Han K Kim HS Park YM Kwon HS Yoon KH. Cholesterol variability and the risk of mortality, myocardial infarction, and stroke: a nationwide population-based study. Eur Heart J. (2017) 38:3560–6. 10.1093/eurheartj/ehx585 29069458 PMC6251576

[16] Bangalore S Breazna A DeMicco DA Wun CC Messerli FH Committee TNTS. Visit-to-visit low-density lipoprotein cholesterol variability and risk of cardiovascular outcomes: insights from the TNT trial. J Am Coll Cardiol. (2015) 65:1539–48. 10.1016/j.jacc.2015.02.017 25881936

[17] Boey E Gay GM Poh KK Yeo TC Tan HC Lee CH. Visit-to-visit variability in LDL- and HDL-cholesterol is associated with adverse events after ST-segment elevation myocardial infarction: a 5-year follow-up study. Atherosclerosis. (2016) 244:86–92. 10.1016/j.atherosclerosis.2015.10.110 26595903

[18] Waters DD Bangalore S Fayyad R DeMicco DA Laskey R Melamed S. Visit-to-visit variability of lipid measurements as predictors of cardiovascular events. J Clin Lipidol. (2018) 12:356–66. 10.1016/j.jacl.2017.12.003 29310989

[19] Chang YH Chang DM Lin KC Hsieh CH Lee YJ. High-density lipoprotein cholesterol and the risk of nephropathy in type 2 diabetic patients. Nutr Metab Cardiovasc Dis. (2013) 23:751–7. 10.1016/j.numecd.2012.05.005 22789808

[20] Kim MK Han K Koh ES Kim HS Kwon HS Park YM. Variability in total cholesterol is associated with the risk of end-stage renal disease: a nationwide population-based study. Arterioscler Thromb Vasc Biol. (2017) 37:1963–70. 10.1161/ATVBAHA.117.309803 28860222 PMC6349220

[21] Takenouchi A Tsuboi A Kitaoka K Minato S Kurata M Fukuo K. Visit-to-visit low-density lipoprotein cholesterol variability is an independent determinant of carotid intima-media thickness in patients with type 2 diabetes. J Clin Med Res. (2017) 9:310–6. 10.14740/jocmr2871w 28270891 PMC5330774

[22] Ng G Boey E Frampton C Richards AM Yeo TC Lee CH. Obstructive sleep apnea is associated with visit-to-visit variability in low-density lipoprotein-cholesterol in patients with coronary artery disease. Sleep Breath. (2017) 21:271–8. 10.1007/s11325-016-1394-0 27502204

[23] Lee SH Han K Cho H Park YM Kwon HS Kang G. Variability in metabolic parameters and risk of dementia: a nationwide population-based study. Alzheimers Res Ther. (2018) 10:110. 10.1186/s13195-018-0442-3 30368247 PMC6204276

[24] Thirumangalakudi L Prakasam A Zhang R Bimonte-Nelson H Sambamurti K Kindy MS. High cholesterol–induced neuroinflammation and amyloid precursor protein processing correlate with loss of working memory in mice. J Neurochem. (2008) 106:475–85. 10.1111/j.1471-4159.2008.05415.x 18410513 PMC3897170

[25] Ehrlich D Humpel C. Chronic vascular risk factors (cholesterol, homocysteine, ethanol) impair spatial memory, decline cholinergic neurons and induce blood–brain barrier leakage in rats *in vivo*. J Neurol Sci. (2012) 322:92–5. 10.1016/j.jns.2012.07.002 22819352 PMC3484398

[26] Ullrich C Pirchl M Humpel C. Hypercholesterolemia in rats impairs the cholinergic system and leads to memory deficits. Mol Cell Neurosci. (2010) 45:408–17. 10.1016/j.mcn.2010.08.001 20696249 PMC2977849

[27] Kivipelto M Helkala EL Laakso MP Hanninen T Hallikainen M Alhainen K. Midlife vascular risk factors and Alzheimer's disease in later life: longitudinal, population based study. BMJ. (2001) 322:1447–51. 10.1136/bmj.322.7300.1447 11408299 PMC32306

[28] Mielke MM Zandi PP Shao H Waern M Ostling S Guo X. The 32-year relationship between cholesterol and dementia from midlife to late life. Neurology. (2010) 75:1888–95. 10.1212/WNL.0b013e3181feb2bf 21068429 PMC2995387

[29] Beydoun MA Beason-Held LL Kitner-Triolo MH Beydoun HA Ferrucci L Resnick SM. Statins and serum cholesterol's associations with incident dementia and mild cognitive impairment. J Epidemiol Community Health. (2011) 65:949–57. 10.1136/jech.2009.100826 20841372 PMC3024452

[30] Anstey KJ Ashby-Mitchell K Peters R. Updating the evidence on the association between serum cholesterol and risk of late-life dementia: review and meta-analysis. J Alzheimers Dis. (2017) 56:215–28. 10.3233/JAD-160826 27911314 PMC5240556

[31] Li G Shofer JB Kukull WA Peskind ER Tsuang DW Breitner JC. Serum cholesterol and risk of Alzheimer disease: a community-based cohort study. Neurology. (2005) 65:1045–50. 10.1212/01.wnl.0000178989.87072.11 16217057

[32] Mielke MM Zandi PP Sjogren M Gustafson D Ostling S Steen B. High total cholesterol levels in late life associated with a reduced risk of dementia. Neurology. (2005) 64:1689–95. 10.1212/01.WNL.0000161870.78572.A5 15911792

[33] Reitz C Tang MX Luchsinger J Mayeux R. Relation of plasma lipids to Alzheimer disease and vascular dementia. Arch Neurol. (2004) 61:705–14. 10.1001/archneur.61.5.705 15148148 PMC2696387

[34] Stewart R White LR Xue QL Launer LJ. Twenty-six-year change in total cholesterol levels and incident dementia: the Honolulu–Asia Aging Study. Arch Neurol. (2007) 64:103–7. 10.1001/archneur.64.1.103 17210816

[35] Notkola IL Sulkava R Pekkanen J Erkinjuntti T Ehnholm C Kivinen P. Serum total cholesterol, apolipoprotein E epsilon 4 allele, and Alzheimer's disease. Neuroepidemiology. (1998) 17:14–20. 10.1159/000026149 9549720

[36] Solfrizzi V Panza F Colacicco AM D'Introno A Capurso C Torres F. Vascular risk factors, incidence of MCI, and rates of progression to dementia. Neurology. (2004) 63:1882–91. 10.1212/01.WNL.0000144281.38555.E3t 15557506

[37] Busse A Bischkopf J Riedel-Heller SG Angermeyer MC. Mild cognitive impairment: prevalence and incidence according to different diagnostic criteria. Results of the Leipzig Longitudinal Study of the Aged (LEILA75+). Br J Psychiatry. (2003) 182:449–54. 10.1192/bjp.182.5.449 12724250

[38] Yoshitake T Kiyohara Y Kato I Ohmura T Iwamoto H Nakayama K. Incidence and risk factors of vascular dementia and Alzheimer's disease in a defined elderly Japanese population: the Hisayama Study. Neurology. (1995) 45:1161–8. 7783883 10.1212/wnl.45.6.1161

[39] Davignon J Ganz P. Role of endothelial dysfunction in atherosclerosis. Circulation. (2004) 109:III27–32. 10.1161/01.CIR.0000131515.03336.f8 15198963

[40] Quinn TJ Gallacher J Deary IJ Lowe GD Fenton C Stott DJ. Association between circulating hemostatic measures and dementia or cognitive impairment: systematic review and meta-analyzes. J Thromb Haemost. (2011) 9:1475–82. 10.1111/j.1538-7836.2011.04403.x 21676170

[41] Chen Z Ichetovkin M Kurtz M Zycband E Kawka D Woods J. Cholesterol in human atherosclerotic plaque is a marker for underlying disease state and plaque vulnerability. Lipids Health Dis. (2010) 9:61. 10.1186/1476-511X-9-61 20540749 PMC2890627

[42] Vedre A Pathak DR Crimp M Lum C Koochesfahani M Abela GS. Physical factors that trigger cholesterol crystallization leading to plaque rupture. Atherosclerosis. (2009) 203:89–96. 10.1016/j.atherosclerosis.2008.06.027 18703195

[43] Mantel-Teeuwisse AK Kloosterman JM Maitland-van der Zee AH Klungel OH Porsius AJ de Boer A. Drug-Induced lipid changes: a review of the unintended effects of some commonly used drugs on serum lipid levels. Drug Saf. (2001) 24:443–56. 10.2165/00002018-200124060-00003 11368251

[44] Constantinides P Booth J Carlson G. Production of advanced cholesterol atherosclerosis in the rabbit. Arch Pathol. (1960) 70:712–24 13695141

[45] Crisby M Nordin-Fredriksson G Shah PK Yano J Zhu J Nilsson J. Pravastatin treatment increases collagen content and decreases lipid content, inflammation, metalloproteinases, and cell death in human carotid plaques: implications for plaque stabilization. Circulation. (2001) 103:926–33. 10.1161/01.CIR.103.7.926 11181465

[46] Bjorkegren JL Hagg S Talukdar HA Foroughi Asl H Jain RK Cedergren C. Plasma cholesterol-induced lesion networks activated before regression of early, mature, and advanced atherosclerosis. PLoS Genet. (2014) 10:e1004201. 10.1371/journal.pgen.1004201 24586211 PMC3937269

