# Supplementary material for: Variability in Total Cholesterol Concentration Is Associated With the Risk of Dementia: A Nationwide Population-Based Cohort Study
Source: Front Neurol. 2019 May 7;10:441. doi: 10.3389/fneur.2019.00441 (PMC6513975; doi:10.3389/fneur.2019.00441)
Supplement: Supplementary file 1 [file Table_1.DOCX]

Supplementary Table 1. Baseline characteristics of subjects according to the total cholesterol variability measured as coefficient of variation (CV)

|  | Q1 | Q2 | Q3 | Q4 | *P*-Value |
| --- | --- | --- | --- | --- | --- |
| N | 32,991 | 32,991 | 32,992 | 32,991 |  |
| Age (years) | 55.6 ± 8.7 | 54.5 ± 8.2 | 54.9 ± 8.4 | 56.8 ± 9.0 | <0.001 |
| Sex (male) (n, %) | 18,620 (56.4) | 19,984 (60.6) | 19,191 (58.2) | 17,032 (51.6) | <0.001 |
| Body mass index (kg/m2) | 23.8 ± 2.8 | 23.8 ± 2.8 | 23.8 ± 2.8 | 23.9 ± 2.9 | <0.001 |
| Systolic BP (mmHg) | 124.5 ± 15.4 | 124.6 ± 15.3 | 124.8 ± 15.4 | 125.6 ± 15.9 | <0.001 |
| Diastolic BP (mmHg) | 77.7 ± 10.1 | 78.0 ± 10.1 | 78.1 ± 10.1 | 78.1 ± 10.2 | <0.001 |
| AST (IU/L) | 25.1 ± 11.3 | 25.4 ± 13.1 | 25.7 ± 14.3 | 27.1 ± 19.6 | <0.001 |
| ALT (IU/L) | 23.6 ± 15.5 | 24.2 ± 18.7 | 24.5 ± 17.9 | 25.6 ± 21.8 | <0.001 |
| GGT (IU/L) | 33.0 ± 36.1 | 35.2 ± 40.3 | 36.0 ± 42.9 | 38.8 ± 55.2 | <0.001 |
| Fasting plasma glucose (mmol/L) | 5.11 ± 0.63 | 5.10 ± 0.64 | 5.11 ± 0.65 | 5.12 ± 0.66 | <0.001 |
| Mean total cholesterol (mg/dL) | 198.8 ± 29.8 | 198.2 ± 29.5 | 197.2 ± 29.8 | 198.8 ± 31.3 | <0.001 |
| Total cholesterol variability |  |  |  |  |  |
| VIM (%) | 8.45 ± 2.84 | 15.07 ± 1.86 | 21.07 ± 2.46 | 34.68 ± 10.64 | <0.001 |
| CV (%) | 4.27 ± 1.41 | 7.62 ± 0.80 | 10.68 ± 1.04 | 17.51 ± 5.07 | <0.001 |
| SD (IU/L) | 8.49 ± 3.09 | 15.10 ± 2.77 | 21.06 ± 3.79 | 34.89 ± 12.48 | <0.001 |
| Current smoker (n, %) | 6,052 (18.3) | 6,798 (20.6) | 6,641 (20.1) | 5,835 (17.7) | <0.001 |
| Alcohol consumption (n, %) | 13,998 (42.4) | 14,861 (45.0) | 14,373 (43.6) | 12,673 (38.4) | <0.001 |
| Regular exercise (n, %) | 3,453 (10.5) | 3,095 (9.4) | 3,091 (9.4) | 3,392 (10.3) | <0.001 |
| Income (lower 10%) (n, %) | 2,335 (7.1) | 2,319 (7.0) | 2,532 (7.7) | 2,848 (8.6) | <0.001 |
| Hypertension (n, %) | 17,636 (53.5) | 18,190 (55.1) | 18,660 (56.6) | 20,110 (61.0) | <0.001 |
| Dyslipidemia (n, %) | 6,627 (20.1) | 8,588 (26.0) | 10,550 (32.0) | 16,634 (50.4) | <0.001 |
| History of myocardial infarction (n, %) | 178 (0.5) | 161 (0.5) | 192 (0.6) | 437 (1.3) | <0.001 |
| Use of anti-hypertensive agent (n, %) | 10,231 (31.0) | 9,833 (29.8) | 10,555 (32.0) | 13,459 (40.8) | <0.001 |
| Use of lipid-lowering agent (n, %) | 2,749 (8.3) | 2,860 (8.7) | 3,638 (11.0) | 7,501 (22.7) | <0.001 |

Abbreviations: AST, aspartate transaminase; ALT, alanine transaminase; BP, blood pressure; GGT, γ-glutamyl transferase; VIM, variability independent of the mean; CV, coefficient of variation; SD, standard deviation

P-value by ANOVA and Chi-square test

Data are expressed as the mean ± SD, or n (%).

Supplementary Table 2. Baseline characteristics of subjects according to the total cholesterol variability measured as standard deviation (SD)

|  | Q1 | Q2 | Q3 | Q4 | *P-*Value |
| --- | --- | --- | --- | --- | --- |
| N | 32,993 | 32,988 | 33,002 | 32,982 |  |
| Age (years) | 55.4 ± 8.7 | 54.5 ± 8.2 | 55.0 ± 8.4 | 56.9 ± 8.9 | <0.001 |
| Sex (male) (n, %) | 18,971 (57.5) | 20,102 (60.9) | 19,042 (57.7) | 16,712 (50.7) | <0.001 |
| Body mass index (kg/m2) | 23.7 ± 2.8 | 23.8 ± 2.8 | 23.9 ± 2.8 | 24.1 ± 2.9 | <0.001 |
| Systolic BP (mmHg) | 124.1 ± 15.4 | 124.4 ± 15.2 | 124.9 ± 15.5 | 126.0 ± 15.9 | <0.001 |
| Diastolic BP (mmHg) | 77.4 ± 10.1 | 77.9 ± 10.0 | 78.2 ± 10.2 | 78.4 ± 10.2 | <0.001 |
| AST (IU/L) | 25.1 ± 11.8 | 25.4 ± 13.6 | 25.9 ± 15.1 | 27.0 ± 18.4 | <0.001 |
| ALT (IU/L) | 23.3 ± 15.7 | 24.1 ± 18.9 | 24.7 ± 18.0 | 25.8 ± 21.3 | <0.001 |
| GGT (IU/L) | 32.3 ± 36.4 | 34.8 ± 40.3 | 36.7 ± 44.4 | 39.2 ± 53.6 | <0.001 |
| Fasting plasma glucose (mmol/L) | 5.09 ± 0.63 | 5.10 ± 0.64 | 5.11 ± 0.65 | 5.14 ± 0.66 | <0.001 |
| Mean total cholesterol (mg/dL) | 189.8 ± 28.7 | 194.0 ± 28.4 | 199.4 ± 28.4 | 209.9 ± 30.9 | <0.001 |
| Total cholesterol variability |  |  |  |  |  |
| VIM (%) | 8.49 ± 2.90 | 15.10 ± 2.02 | 21.07 ± 2.66 | 34.61 ± 10.70 | <0.001 |
| CV (%) | 4.42 ± 1.61 | 7.78 ± 1.40 | 10.72 ± 1.85 | 17.17 ± 5.36 | <0.001 |
| SD (IU/L) | 8.20 ± 2.71 | 14.77 ± 1.60 | 20.96 ± 2.12 | 35.61 ± 11.88 | <0.001 |
| Current smoker (n, %) | 6,126 (18.6) | 6,812 (20.6) | 6,608 (20.0) | 5,780 (17.5) | <0.001 |
| Alcohol consumption (n, %) | 14,113 (42.8) | 14,951 (45.3) | 14,263 (43.2) | 12,578 (38.1) | <0.001 |
| Regular exercise (n, %) | 3,416 (10.4) | 3,046 (9.2) | 3,145 (9.5) | 3,424 (10.4) | <0.001 |
| Income (lower 10%) (n, %) | 2,274 (6.9) | 2,300 (7.0) | 2,545 (7.7) | 2,915 (8.8) | <0.001 |
| Hypertension (n, %) | 17,184 (52.1) | 180,21 (54.6) | 18,856 (57.1) | 20,535 (62.3) | <0.001 |
| Dyslipidemia (n, %) | 4,262 (12.9) | 6,571 (19.9) | 10,695 (32.4) | 20,871 (63.3) | <0.001 |
| History of myocardial infarction (n, %) | 182 (0.6) | 163 (0.5) | 201 (0.6) | 422 (1.3) | <0.001 |
| Use of anti-hypertensive agent (n, %) | 10,007 (30.3) | 9,879 (29.9) | 10,565 (32.0) | 13,627 (41.3) | <0.001 |
| Use of lipid-lowering agent (n, %) | 2,161 (6.5) | 2,482 (7.5) | 3,464 (10.5) | 8,641 (26.2) | <0.001 |

Abbreviations: AST, aspartate transaminase; ALT, alanine transaminase; BP, blood pressure; GGT, γ-glutamyl transferase; VIM, variability independent of the mean; CV, coefficient of variation; SD, standard deviation

P-value by ANOVA and Chi-square test

Data are expressed as the mean ± SD, or n (%).
